# Antigen-Specific T Cells and SARS-CoV-2 Infection: Current Approaches and Future Possibilities

**DOI:** 10.3390/ijms232315122

**Published:** 2022-12-01

**Authors:** Zuzana Nova, Tomas Zemanek, Norbert Botek

**Affiliations:** 1 Lambda Life a.s., Levocska 3617/3, 851 01 Bratislava, Slovakia; 2 GAMMA-ZA s.r.o., Kollarova 8, 911 01 Trencin, Slovakia; 3 Department of Oncology, Faculty of Medicine and Dentistry, Palacky University Olomouc, University Hospital Olomouc, Hnevotinska 976/3, 77900 Olomouc, Czech Republic; 4 Department of Laboratory Medicine, Faculty of Health Care and Social Work, Trnava University, Univerzitne Namestie 1, 918 43 Trnava, Slovakia

**Keywords:** COVID-19, severe acute respiratory syndrome coronavirus 2, virus-specific T cells, cellular therapies, immunotherapy

## Abstract

COVID-19, a significant global health threat, appears to be an immune-related disease. Failure of effective immune responses in initial stages of infection may contribute to development of cytokine storm and systemic inflammation with organ damage, leading to poor clinical outcomes. Disease severity and the emergence of new SARS-CoV-2 variants highlight the need for new preventative and therapeutic strategies to protect the immunocompromised population. Available data indicate that these people may benefit from adoptive transfer of allogeneic SARS-CoV-2-specific T cells isolated from convalescent individuals. This review first provides an insight into the mechanism of cytokine storm development, as it is directly related to the exhaustion of T cell population, essential for viral clearance and long-term antiviral immunity. Next, we describe virus-specific T lymphocytes as a promising and efficient approach for the treatment and prevention of severe COVID-19. Furthermore, other potential cell-based therapies, including natural killer cells, regulatory T cells and mesenchymal stem cells are mentioned. Additionally, we discuss fast and effective ways of producing clinical-grade antigen-specific T cells which can be cryopreserved and serve as an effective “off-the-shelf” approach for rapid treatment of SARS-CoV-2 infection in case of sudden patient deterioration.

## 1. Introduction

Coronavirus disease 2019 (COVID-19) caused by severe acute respiratory syndrome coronavirus 2 (SARS-CoV-2) has become one of the main causes of death worldwide, and the pandemic continues despite the development of highly efficacious vaccines. An important hallmark of severe infection is continued T cell depletion, resulting in uncontrolled inflammatory response with massive cytokine and chemokine release (so-called “cytokine storm”) that can progress to systemic inflammation, multi-organ damage and even death [1,2,3]. T cells and virus-specific T cells are essential to protect organisms against viral infection. Patients with a susceptible immune system may therefore be at great risk of fatal outcomes following SARS-CoV-2 infection. Indeed, elevated hospitalization and mortality rates have been described in immunocompromised patients [4,5,6,7]. Remy et al. [8] have recently suggested that immune depression, rather than immune hyperactivation, could be responsible for the clinical pathology of severe COVID-19, although both T cell hyperactivation and exhaustion have been reported in COVID-19 [9].

To date, there are no approved direct antiviral therapies to treat COVID-19 patients, and current curative strategies focus only on supportive care and treatment of disease symptoms (European Medicines Agency, EMA; U.S. Food and Drug Administration, FDA). Generally, many therapeutic options for viral diseases have severe adverse effects, could develop resistance or fail to ensure long-term protection. Therefore, adoptive cell therapy using virus-specific T cells (VSTs) has been used as an alternative treatment strategy, emerging as a promising approach due to its minimal toxicity [10,11,12,13]. Donor-derived T cells with specificity for adenovirus (AdV), cytomegalovirus (CMV), Epstein-Barr virus (EBV), BK virus or human herpesvirus have been used in previous studies to prevent or treat viral infections in the settings of immunity errors or after transplantation, demonstrating its safety and efficacy [10,12,14,15,16,17,18]. In addition to the reconstitution of antiviral immunity after treatment, it has been shown that VSTs persist for years after infusion and thus can provide long-term protection [19,20]. Data on SARS-CoV-1 infection have shown that T cell responses can last up to 17 years, while antibodies often weaken 1–2 years after disease [21]. The potential application of immunotherapy could also be relevant to patients at high risk of severe COVID-19, especially immunocompromised individuals with T cell deficiency or those receiving immunosuppressive therapies. This approach may decrease initial viral load [22], increase early bystander CD8^+^ T cell activation [23] and ensure adequate early type I interferon response [24,25], leading to enhanced viral clearance and prevention of severe disease development. Cytokine release syndrome, a major complication of COVID-19 [26], has been reported also with AdV, CMV or EBV infections [11,27,28], where VST therapy has been successfully used to treat many patients suffering from severe infections, with minimal complications [10,11,12,13]. Adoptively infused SARS-CoV-2-specific T cells (CSTs) are believed to target and kill virus-infected myeloid cells, resulting in breaking of the vicious cycle which drives the cytokine storm [29]. Infusion of CSTs could therefore be a life-saving strategy for severe SARS-CoV-2-infected patients.

## 2. Mechanism of Cytokine Storm Development in COVID-19

Once the virus enters the body, viral components are recognized by Toll-like receptors (TLRs) expressed on the surface of antigen-presenting cells (APCs), which rapidly induce anti-viral responses via the production of pro-inflammatory cytokines and type I interferons in order to clear infection [30]. Under normal conditions, innate (natural killer, NK) and adaptive (T cells) immune responses tend to cause an apoptosis of APCs and thus prevent unnecessary hyperactivation. However, any alterations in the lymphocyte activity caused by SARS-CoV-2 infection leads to the inactivation of both NK and cytotoxic T cells and their inability to kill APCs, resulting in dysregulation of immune balance, recruitment of more inflammatory cells, abnormal release of cytokines at the site of infection and induction of cytokine storm. Dysregulation of T cells induced by SARS-CoV-2 infection is described in more detail in the next chapter. The most commonly affected organ in COVID-19 patients are the lungs, manifesting as severe pneumonia, pulmonary edema and acute respiratory distress syndrome (ARDS) [31,32]. Subsequently, impaired vascular permeability due to cytokine storm further leads to cytokine release into the systemic circulation, damage of other organs and even death [3]. However, significant differences in clinical manifestation of COVID-19 between adults and children have been observed. SARS-CoV-2 infection of children is mostly asymptomatic or leads to a mild illness. Some pediatric patients can develop a post-infectious multisystem inflammatory syndrome in children (MIS-C), but these cases and mortality are rare. The immunological basis and immune mechanisms of differing disease severity and outcomes between children and adults have not been yet sufficiently characterized, but age-dependent factors and less comorbidities have been suggested to modulate antiviral immune response. Nonetheless, this issue is beyond the scope of this review and was described in more detail by others [33,34,35,36].

**Figure 1:**
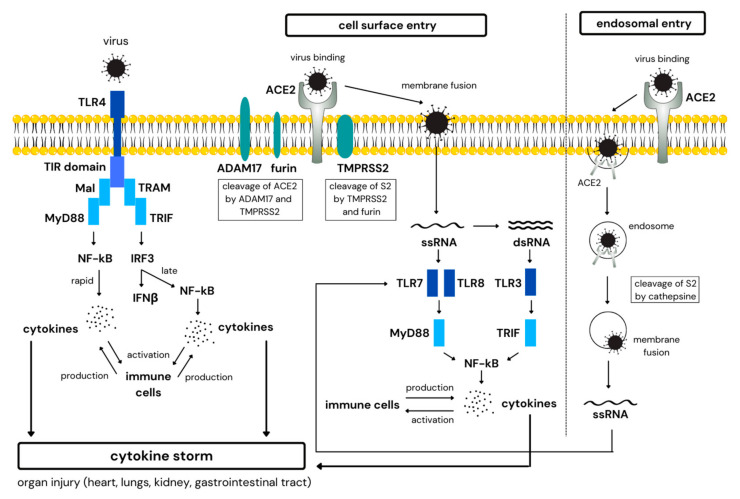
Mechanism of cytokine storm development in COVID-19. ACE2—angiotensin-converting enzyme 2, ADAM17—A disintegrin and metalloproteinase 17, dsRNA—double-stranded RNA, IFNβ—interferon beta, IRF3—interferon regulatory factor 3, MAL—MyD88 adaptor-like protein, MyD88—myeloid differentiation primary response protein 88, NF-κB—nuclear factor kappa B, ssRNA—single-stranded ribonucleic acid, TIR—Toll/interleukin receptor, TLR—Toll-like receptor, TMPRSS2—transmembrane serine protease 2, TRAM—TRIF-related adaptor molecule, TRIF—TIR domain-containing adaptor-inducing interferon-β.

Viral components are recognized by Toll-like receptors (TLRs). It has been suggested that cell surface TLR4 and intracellular TLR3, TLR7 and TLR8 contribute to antiviral responses against SARS-CoV-2 infection (Figure 1) [37,38]. TLR4 recognizes and binds SARS-CoV-2 spike (S) glycoprotein [39,40], TLR7/8 detects viral single-stranded ribonucleic acid (ssRNA) and ligand for TLR3 is a viral double-stranded RNA ribonucleic acid (dsRNA) [38,41]. TLRs trigger two signal transduction pathways, (i) the myeloid differentiation primary response protein 88 (MyD88)-dependent with MyD88 adaptor-like protein (MAL), leading to activation of nuclear factor kappa B (NF-κB) pathway and rapid production of pro-inflammatory cytokines such as interleukin (IL)-6, IL-1β or tumor necrosis factor α (TNF-α), and (ii) the MyD88-independent pathway mediated through Toll/interleukin-1 receptor (TIR)-domain-containing adaptor protein (TRIF) and TRIF-related adaptor molecule (TRAM), leading to release of type I interferons (IFN-I; IFN-α/β) through the induction of interferon regulatory factor-3 (IRF-3), and late NF-κB activation. TLR4 mediates signals through both pathways, TLR3 through TRIF and TLR7/8 utilize the MyD88 pathway. Both pathways contribute to elevated levels of cytokines in the body [3,38,42,43]. IFN-I response is critical to combat viral infections. However, it is quickly and selectively abrogated by SARS-CoV-2 via different mechanisms [44,45]. This inhibition ability is associated with clinical severity of the disease [46]. Clinical studies have demonstrated that coronaviruses are able to evade innate immunity during the first 10 days of infection, allowing elevated virus replication, which corresponds to a period of widespread inflammation and steadily increasing viral load [47,48]. Indeed, several groups have reported remarkably reduced IFN-I response in severe COVID-19 patients [24,25,49]. This is accompanied by an overwhelming production of pro-inflammatory cytokines, accumulation of pathogenic monocyte-macrophages, and suboptimal T cell response, leading to hyperinflammation and cytokine storm [44,50]. In addition to the direct role of TLR4 signaling in mediating the immunopathogenesis, TLR4 activation resulting from its interaction with spike (S) protein also increases cell surface expression of key SARS-CoV-2 receptor angiotensin-converting enzyme 2 (ACE2), which in turn augments viral load as well as hyperinflammatory events [51].

The interaction between ACE2 and S protein of SARS-CoV-2 is responsible for viral entry to the host cells (Figure 1) [52,53,54,55]. Additionally, S protein cleavage by cellular transmembrane serine protease 2 (TMPRSS2) is required to allow fusion of viral and cellular membranes [52,55]. In the respiratory tract, ACE2 and TMPRSS2 are co-expressed in alveolar epithelial type II (ATII) cells [56], alveolar macrophages [54,57,58] and nasal goblet secretory cells [56]. Another enzyme involved in the viral entry process is furin, which is highly expressed in lung tissue [54,59,60]. ACE2, TMPRSS2 and furin triple expression has been found in lung macrophages [57]. If ACE2-virus complex could not find TMPRSS2 or the target cell does not express enough TMPRSS2, the virus is internalized into the endolysosomes, where it is cleaved by cathepsins (Figure 1). After the fusion of viral and cellular membranes, viral ribonucleic acid (RNA) is released into the cytoplasm and can be replicated into dsRNA [55]. ssRNA and dsRNA are then recognized by TLR7/8 and TLR3, respectively, and trigger pro-inflammatory pathways as mentioned above [38,41].

**Figure 2:**
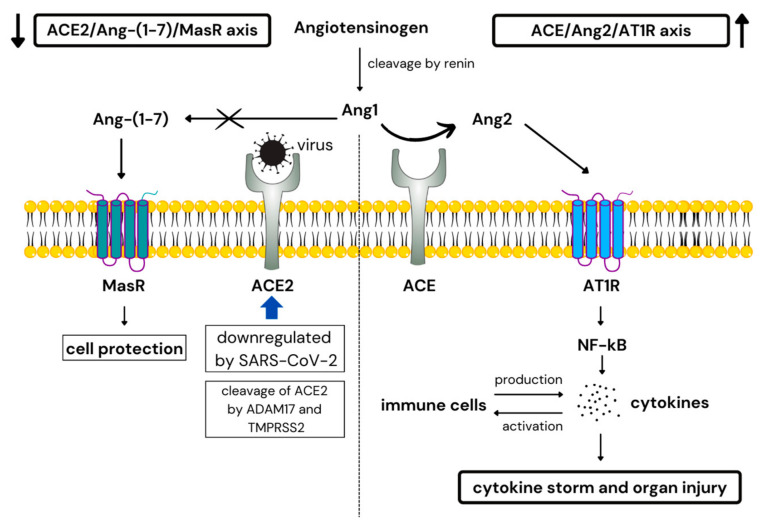
ACE/Ang2/AT1R and ACE2/Ang-(1-7)/MasR axis during COVID-19. ACE—angiotensin-converting enzyme, ACE2—angiotensin-converting enzyme 2, ADAM17—A disintegrin and metalloproteinase 17, Ang—angiotensin, AT1R—angiotensin II receptor type 1, MasR—Mas receptor, NF-κB—nuclear factor kappa B.

ACE2 has been described as an important cell-protective component as it acts like a negative regulator of the renin-angiotensin pathway (RAS), thus having an anti-inflammatory and anti-fibrotic effect. SARS-CoV-2 triggers ACE2 downregulation via its cleavage by TMPRSS2 and A disintegrin and a metalloproteinase 17 (ADAM17) enzyme, leading to imbalance between ACE2 and its counterpart ACE [61,62,63,64]. ACE upregulates the level of angiotensin II (Ang2), which excessively binds to angiotensin II type-1 receptor (AT1R) and triggers inflammatory response. Ang2 interacts with both tissue-resident and immune cells. Activated tissue-resident cells produce pro-inflammatory factors, such as NF-κB, TNF-α, IL-6, IL-1β, IFN-γ, prostaglandins and vascular endothelial cell growth factor (VEGF). Moreover, Ang2 promotes immune cell recruitment into the site of injury and stimulates the production of cytokines and chemokines, resulting in amplification of inflammatory cycle and tissue damage (Figure 2) [62,65,66]. Additionally, Ang2 upregulates the expression of TLR4, leading to stimulation of NF-κB and induction of pro-inflammatory cytokines [67]. Highly elevated levels of cytokines, especially IL-6 increase vascular permeability, leading to release of pro-inflammatory cytokines to the systemic circulation, which further contribute to the cytokine storm and subsequent multi-organ failure [26,66].

Compared to adults, the lower expression of ACE2 has been described in nasal and bronchial tissue of children [68,69]. Also, other research groups have suggested that ACE2 expression in nasal and lung epithelium increases with age, which could be one of many possible explanations for the difference in COVID-19 severity observed between children and adults [70,71]. Moreover, expression of TMPRSS2 rises with age, and is significantly higher in the airway epithelium of adults compared to infants [34,71,72].

## 3. Dysregulation of T Cells in COVID-19

**Figure 3:**
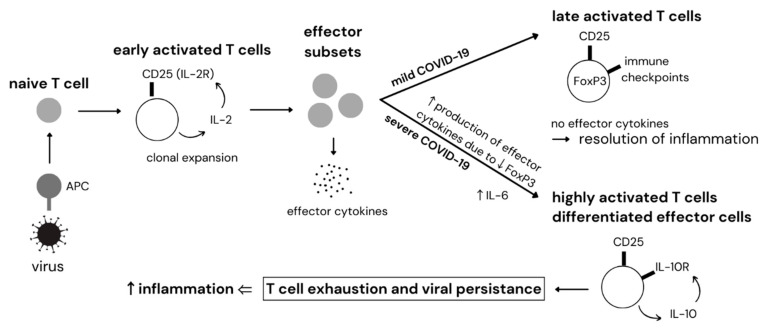
Dysregulation of T cells in COVID-19. APC—antigen-presenting cell, CD25—cluster of differentiation 25, FOXP3—forkhead box-P3, IL—interleukin, IL-2R—interleukin 2 receptor, IL-10R—interleukin 10 receptor.

T cells have a key role in immune reactions during COVID-19. CD4^+^ T helper cells regulate the development of high-affinity neutralizing antibodies, B cell differentiation into memory and long-lived antibody secreting cells and also CD8^+^ T cell activation through provision of soluble mediators and co-stimulation. CD8^+^ cytotoxic T lymphocytes secrete antiviral cytokines, such as IFN-γ and TNF-α, and directly kill virus-infected cells [9,73]. During a primary immune response, APCs identify and present foreign antigens to T cells, which recognize them through their T cell receptor (TCR) [74,75]. Once viral particles are recognized, the TCR signaling pathway triggers T cell activation, proliferation and differentiation into effector subsets. Initially, the expression of CD25 (cell surface marker of IL-2 receptor α-chain) [76,77] and level of IL-2 is rapidly upregulated in early activated T cells [78,79], creating a positive feedback loop for T cell activation and acquiring effector functions in order to suppress infection. The subsequent fate of activated cells differs in mild and severe SARS-CoV-2 infection (Figure 3). In mild COVID-19 patients, IL-2 drives differentiation into regulatory T cells (Tregs) expressing transcription factor forkhead box-p3 (FOXP3), which is crucial for maintaining immune tolerance and immune system homeostasis [80]. Simultaneously, the expression of immune checkpoint molecules, such as cytotoxic T-lymphocyte–associated antigen 4 (CTLA-4) is increased, providing a negative feedback regulation. This leads to conversion of early activated T cells into the suppressive state and resolution of inflammation [81,82]. Following antigen clearance, most activated effector T cells die by apoptosis, but a subset persists and differentiate into long lived-memory cells [83]. In severe COVID-19 patients, T cell expression of FOXP3 is repressed, likely due to high levels of pro-inflammatory cytokines, especially IL-6, which has emerged as a key inducer of the cytokine storm [84,85]. Therefore, highly activated effector T cells continuously produce multiple pro-inflammatory cytokines and furin [79,86,87]. Furin can further enhance SARS-CoV-2 entry to host cells, as mentioned above. Prolonged immune activation alters the lymphocyte secretome. Activated T cells are differentiated into a CD25^+^ IL-10R+ phenotype, resulting in production of IL-10 rather than IL-2 [88]. Under these conditions, the IL-10 regulatory loop is created and amplified, resulting in loss of CD4^+^ and CD8^+^ cell functions and inability to respond against pathogens efficiently [89,90,91,92]. A recent study showed that dramatic early IL-10 elevation may contribute to COVID-19 severity by promoting T cell exhaustion [93]. Furthermore, inhibitory molecules are overexpressed. Programmed cell death 1 receptor (PD-1) [1,83,94,95], CTLA-4 [95,96], T cell immunoglobulin and mucin-domain containing-3 (Tim-3) [1,97], lymphocyte-activation gene 3 (LAG-3) [95], B- and T-lymphocyte attenuator (BTLA) [98] and inhibitory receptor NKG2A [99] have been identified as markers of chronic activation, inhibition and exhaustion in both CD4^+^ and CD8^+^ T cells. Because of T cell inability to clear pathogen, viral load and antigen exposure time increase, further contributing to exhaustion [100]. Several studies have claimed that CD8^+^ T cells primed during chronic infections are able to recover and differentiate into memory T cells after adoptive transfer into uninfected mice in the course of the first one to three weeks of infection, while longer exposure to infection results in irreversible exhaustion [101,102,103]. It has been shown that frequency of multi-functional CD4^+^ T cells and non-exhausted CD8^+^ T cells were significantly lower in severe COVID-19 cases compared with mild group and healthy controls, whereas the number of non-functional subsets significantly increased [1,104]. Hence, SARS-CoV-2 infection may break down antiviral immunity at an early stage of disease. However, the exact mechanism is not fully understood yet. Moreover, SARS-CoV-2 can directly infect T cells via ACE2 receptor expressed on T cells [105], which results in T cell death and potentially contributes to reduced T cell number and immune dysregulation observed in COVID-19 illness. Several studies have claimed that severe SARS-CoV-2 infection is associated with marked reduction of all lymphocyte subsets (lymphopenia) that correlate with morbidity and mortality [1,94,106,107]. Therefore, immune reconstitution, marked by increased T cell number, is crucial for COVID-19 patients.

The situation is slightly different in pediatric patients. Studies have shown that only 3.5–5.2% of children with COVID-19 had lymphopenia, and the majority had normal leukocyte numbers [33,108,109]. Furthermore, differences in interferon signaling have been investigated in children and adults with COVID-19. Children can develop more robust interferon response after TLR stimulation [35,110]. It has been reported that increased production of IFN-γ during an early immune response can result in a more rapid clearance of viral infection [33]. It has been also shown that SARS-CoV-2-specific T cell responses differ in infected adults and children. Acute and memory CD4^+^ T cell reactivity to structural SARS-CoV-2 proteins increase with age, whereas CD8^+^ T cell responses increase with time post-infection. Moreover, CD4^+^ T cells are targeting primarily structural proteins in adults, while pediatric CD4^+^ T cells significantly target non-structural proteins in open reading frame (ORF) 1ab [34]. Some of them are able to block the host interferon response [111], which could contribute to the age-associated decline of IFN response to infection by SARS-CoV-2. Likewise, children’s adaptive immune system differs from adults. Thus, the immune system of pediatric patients is less likely to overreact to SARS-CoV-2 infection. Stronger T-cell response to SARS-CoV-2 spike protein and a higher neutralizing antibody count were observed in adults compared to children [33]. Taken together, milder adaptive immune response, but more robust innate response in children may prevent a cytokine storm, possibly eliminating an unwanted overreaction to the virus. Despite that, immunological differences with adults are not fully understood, and further research is still needed to discern the protective role of T cells in children with COVID-19.

## 4. SARS-CoV-2-Specific T Cell Therapy for the Treatment and Prevention of Severe COVID-19 Infection

Several recent studies have pointed out a link between severe COVID-19 and insufficient early innate immune responses to SARS-CoV-2, leading to weak or delayed adaptive response [107,112]. In primary (SARS-CoV) infection, CD4^+^ T cells settle virus control and affect disease progression. Aged BALB/c mice with CD4^+^ T cell depletion experienced upregulated cytokine production, protracted SARS-CoV clearance from the lungs, decline in neutralizing antibody titers and pulmonary influx of lymphocytes [113]. Nucleocapsid (N)-specific airway memory CD4^+^ T cells produced via intranasal vaccination successfully protected against lethal disease via accelerated production of IFN-γ. Airway depletion of CD4^+^ cells and IFN-γ lowered survival rate and manifested as elevated pathological changes in the lungs [114]. Similar results were also described in other animal models [115,116,117]. Furthermore, murine studies of pathogenic coronavirus infection largely demonstrated a protective role for T cells and suggested that SARS CoV-2-specific CD4^+^ and CD8^+^ T cells will control virus replication and moderate the pathology associated with COVID-19 [118].

Analysis of bronchoalveolar lavage fluid (BALF) from COVID-19 patients revealed decreased clonal T cell expansion and suboptimal presence of CSTs in the lungs of those with severe or fatal disease [119,120]. Moreover, depletion of CD8^+^ T cells in hematologic malignancy patients with impaired humoral responses to SARS-CoV-2 has been associated with poor outcomes [121]. Indeed, lymphopenia is a typical profile in patients with COVID-19, especially in severe cases [9]. It has been shown that the numbers of total T cells were negatively correlated to serum level concentrations of IL-6, IL-10, and TNF-α [1]. Thus, enhancing immune reconstitution, especially CSTs, is the most crucial process which takes place in treatment of COVID-19 patients. In a convalescent patient cohort, CD4^+^ and CD8^+^ CSTs were detected in peripheral blood mononuclear cells (PBMCs) from more than 90% and 70% of individuals, respectively. Presence of CSTs positively corelated with recovery and decreased illness severity [73,122,123,124,125]. These findings indicate the importance of T cell responses, particularly CSTs, for effective viral clearance in patients with acute infection. Studies have shown suboptimal responses of immunocompromised patients to vaccination [126,127,128,129,130,131]. Booster vaccines are required in this population, but it is unclear how durable will be their protective immunity compared with immunocompetent individuals. Adoptive cellular therapy with CSTs could therefore be a promising treatment or prophylactic strategy leading to improved clinical outcomes in patients with or at high risk for COVID-19. A review of VSTs given to 36 patients with primary immunodeficiency disorders reported excellent responses to VSTs both for prophylaxis (81% of patients protected from viral reactivation) and treatment (response rates 76–100% depending on the virus) [132]. Considering long-lasting T cell responses against SARS-CoV-1 [124,133] and Middle East respiratory syndrome coronavirus (MERS-CoV) [134], specific SARS-CoV-2 memory T cells are believed to sustain for a longer period with the ability to clear viral re-infection. Very recently, it was reported that T cell memory persists for 8 [123] to 10 months [135] after primary SARS-CoV-2 infection, which further supports the potential of CSTs to maintain durable protection against severe COVID-19 despite humoral immunity declines [136]. Several clinical trials are ongoing to determine the effect of CSTs for the treatment or prevention of SARS-CoV-2 infection (Table 1).

## 5. Large-Scale Production of SARS-CoV-2-Specific T-Cells

Production of VSTs for clinical use consists of the selection of potential donor, verification of his specific T cell frequencies and prediction of T cell enrichment efficiency, subsequent VSTs ex vivo expansion from donor’s PBMCs and final quality control. T cell donor selection is based on human leukocyte antigen (HLA) type, viral serostatus and presence of VSTs in peripheral blood. It has been reported that SARS-CoV-2-specific CD4^+^ and CD8^+^ T cells are capable of activating and proliferating ex vivo for at least 10 months after infection [135]. Direct selection of VSTs uses major histocompatibility complex (MHC)–antigen multimers or cytokine capture system (CCS; IFN-γ capture system) after cell restimulation with viral antigens [137,138,139]. While the knowledge of immunodominant HLA-restricted peptides is required for MHC multimer technology [137,140], CCS does not have any HLA restrictions and can be performed with recombinant proteins or overlapping peptide pools spanning the entire viral proteome, enabling the generation of CD4^+^ and CD8^+^ T cells specific to multiple epitopes [138,141,142]. Antigen-specific T cells prepared by the IFN-γ CCS have been classified as an advanced-therapy medicinal product by the Committee for Advanced Therapies of the European Medicines Agency [143]. Briefly, suitable donor VSTs isolated from PBMCs are cultivated in the presence of viral antigens and growth-enhancing cytokines, such as IL-4 + IL-7 [15], IL-1 + IL-6 + IL-23 [144], IL-2 + IL-4 + IL-7 or IL-2 + IL-7 + IL-15 [29] for 10 to 12 days [139]. Such combinations of cytokines can promote the survival of activated T cells by upregulation of anti-apoptotic molecules and support the retention of their central memory phenotype [15,144]. Subsequently, IFN-γ+ virus-specific T cells are captured by magnetic cell sorting enrichment processes [138]. Several research groups have successfully generated CSTs using CliniMACS CCS [145,146,147,148,149].

Optimal HLA matching strategy is still under study. In the case of allogeneic hematopoietic stem cell transplantation (HSCT), seropositive donors can usually also provide T cell donation. However, some of them may not be available or have an insufficient amount of memory T cells in their peripheral blood despite seropositivity. Moreover, granulocyte colony-stimulating factor (G-CSF) treatment usually used for mobilization of PBMCs in healthy volunteers before stem cell donation has been shown to have long-term negative effects on T cell functional activity [150,151,152]. In general, adoptive transfer of donor-derived VSTs is highly patient-specific and requires virus-immune donors, which has emerged as a barrier that hinders broad implementation of this therapy. Thus, partially HLA-matched VSTs have been investigated as an alternative therapeutic option [13,153,154,155,156]. Such products can be cryopreserved and stored, allowing for rapid treatment of infection if necessary. Indeed, the safety and efficacy of this approach has been determined in several studies [19,154,157,158,159] and was shown to not induce de novo graft-versus-host disease (GvHD) despite in vitro recognition of recipient HLA molecules [157]. Available data indicate that at least one shared HLA allele is necessary to mediate antiviral T cell immunity [160]. Leung and colleagues [145] successfully prepared CSTs from six convalescent COVID-19 patients using IFN-γ CCS and SARS-CoV-2-specific PepTivators (peptide pools consisting mainly of 15-mer sequences with 11 amino acids overlap). They hypothesized 88 to 99% probability that the recipient will share at least one HLA allele with one of the six donors, depending on ethnic background. The peptide pool used in this study consists of 88 peptides with a length of 9–22 amino acids originating from structural and non-structural proteins. Sixty-three of these peptides are restricted to HLA class I, and 25 peptides are restricted to HLA class II. Generally, the selected peptides allow a broad HLA coverage throughout the Caucasian population and stimulate both CD4^+^ and CD8^+^ T cell subpopulations. HLA-A, B and DR alleles represented in this peptide pool are indeed present in >95%, >85% and >90% of the donors in the alloCELL registry (http://www.alloCELL.org (accessed on 9 September 2022)). Moreover, the majority of these peptides are identical in wild-type and presently circulating variants of concern, including Delta and Omicron. It is assumed that future emerging variants will also be adequately covered by used PepTivator SARS-CoV-2 Select, making it suitable for donor selection and subsequent clinical-scale manufacturing of CSTs [145,149]. Interestingly, Tzannou et al. [161] successfully prepared a small bank of VSTs from strategically selected third-party donors based on their HLA profile and immunity to the target of interest and thus provided potential treatment to a large racially and ethnically diverse patient population. The effect of partially HLA-matched CSTs in high-risk patients hospitalized with COVID-19 was described in clinical trial NCT04401410. One of them developed cytokine release syndrome symptoms but recovered, and the infection was successfully cleared in the remaining three patients after CST infusion [162]. However, because of the missing control group, the extent of the CST therapy cannot be clearly defined, and this trial was terminated due to insufficient number of recruited patients. Thus, the use of third-party donors together with quick and effective isolation methods (IFN-γ capture assay, MHC multimer technology) could be a promising approach to supply antigen-specific effector cells for treatment of potentially life-threatening infections in immunocompromised patients.

CST products can be generated not only from virus-experienced but also virus-naive donors, as SARS-CoV-2-specific T cell responses were observed in some unexposed individuals, likely due to pre-existing cross-reactivity with common seasonal coronaviruses [73,146,163,164,165]. The pool of potential donors can be also enriched with individuals vaccinated against SARS-CoV-2 [166,167,168], whose specific T cell responses have been proven effective against other virus variants [169,170]. The generation of CSTs from vaccinated donors on a clinically relevant scale was successfully demonstrated for the first time by Taborska et al. [171]. Such cross-reactivity of generated CSTs against new viral strains is essential for its therapeutic use. It has been suggested that most of the memory CD4^+^ T cell response is conserved against SARS-CoV-2 variants of concern [170]. Indeed, the vast majority of T cell epitopes identified in recovering patient CSTs appeared to be conserved in SARS-CoV-2 genome modifications [15,172,173,174,175]. The strong cross-reactivity of CSTs against circulating variants has been detected in several studies [166,168,169,176]. Moreover, CSTs from convalescent patients were able to respond to viral strains partially resistant to vaccine-induced humoral immunity [177,178,179]. However, other research groups analyzed the response of CD8^+^ T cells and have found that the ability of some mutant epitopes to bind to certain HLAs is decreased [180,181]. Thus, it is important to ensure that CSTs manufactured for clinical practice are effective against new emerging SARS-CoV-2 variants.

Because of the hyperinflammatory nature of COVID-19, many patients with severe disease receive a systemic corticosteroid therapy, which may improve clinical outcomes [182,183]. However, commonly used dexamethasone is highly lymphocytotoxic [184,185]. This could significantly limit the efficacy of CST treatment as corticosteroids are highly likely to induce apoptosis of adoptively transferred T cells. Therefore, the question arises as to how effective T-cell therapy would be in the setting of pneumonia or ARDS. Very recently, Basar et al. [29] proposed an efficient strategy to inactivate the glucocorticoid receptor gene (NR3C1) in cytotoxic T lymphocytes using CRISPR-Cas9 gene editing. Such modified cells retained viability and effector functions in the presence of dexamethasone in vitro. This approach could make CST application clinically feasible even when simultaneous immunosuppressive pharmacotherapy is required.

## 6. Other Potential Cell-Based Therapies for COVID-19

In addition to antigen-specific T cells, therapies using NK cells, Tregs or mesenchymal stem cells (MSCs) could potentially have a beneficial effect for COVID-19 patients. NK cells are an essential part of antiviral defense, as evidenced by inborn errors of NK cell development that led to increased viral susceptibility [186,187]. NK cell exhaustion and their low numbers in the blood are common in patients with moderate and severe COVID-19 disease [188,189,190], which may result in decreased clearance of infected and activated cells, and elevation of inflammation markers. Indeed, inverse correlations between NK cell counts and serum levels of IL-6 [189] and interleukin-2 receptor alpha [191] have been reported. The potential role of NK cells in fighting viral infections, including COVID-19, has been mentioned in several publications [192,193,194,195]. It turns out that adoptive transfer of highly activated NK cells at the beginning of the disease might help to boost innate and adaptive immunity, improving survival and reducing disease progression rates in COVID-19 patients [196]. Furthermore, Ma et al. [197] proved that NK cells modified with a chimeric antigen receptor (CAR) using single-chain variable fragment (scFv) domain of S309, a monoclonal antibody targeting SARS-CoV-2 S glycoprotein, successfully killed spike-expressing target cells and were also cross-reactive with other spike variants. There are several clinical trials evaluating safety and efficiency of immunotherapy with allogeneic NK cells as an adjunctive treatment for SARS-CoV-2 infection (NCT04900454, NCT04280224, NCT04365101, NCT04634370, NCT04363346, NCT04578210) and one trial focused on CAR-modified NK cells targeting the S protein of SARS-CoV-2 (NCT04324996).

The therapeutic potential of ex vivo expanded MSCs and Tregs in ARDS is based on their immunomodulatory and tissue reparative properties. Promising results of both treatments have been shown in preclinical and early clinical trials [198,199,200,201,202,203]. MSCs are unlikely to be infected by SARS-CoV-2 as they do not express ACE2 and TMPRSS2 receptors [204,205,206]. As a result, they may be used for treatment of affected tissues without being destroyed by the virus. In addition to regenerative potency [207], MSCs are able to inhibit the overactivated immune system [208,209] and also repair defective immune function [210]. Importantly, they can suppress cytokine production and mitigate cytokine storm [204,211,212]. Avoiding the cytokine storm may be the key for the treatment of COVID-19 patients. Beneficial properties of MSCs are mediated by direct interaction with immune cells and secretion of paracrine factors, such as keratinocyte growth factor, hepatocyte growth factor or angiopoietin-1, which have been linked with lung protection [204,213,214,215]. The advantage of MSCs is their low surface expression of HLA antigens, which allows for allogeneic infusion without HLA matching [216,217,218]. MSCs were used in several pilot studies for treatment of severe COVID-19, demonstrating their efficiency by patient clinical improvement, better oxygenation and decreased inflammatory markers (CRP, IL-6, TNF-α, procalcitonin) with no observed adverse effects [204,219,220,221,222]. Promising preliminary results of MSC efficacy were also shown in randomized controlled trials [203,223,224,225]. Interestingly, MSC-derived exosomes appear to be a promising therapeutic alternative for severe COVID-19 as they are easy to produce and supply, having comparable therapeutic efficacy to MSCs administration [226,227].

It has been shown that Tregs are able to hinder activation of innate as well as adaptive immune cells by inhibitory surface molecules (LAG-3; CTLA-4) and secretion of immunosuppressive cytokines (IL-10, IL-35, transforming growth factor beta-TGF-β), which points to their importance in prevention of inflammation-induced tissue damage during acute infections and tissue reparation [200,228,229,230]. Peripheral Treg count is prominently reduced in severely ill COVID-19 patients [231,232,233,234], which might be one of the reasons for the immune system hyperactivation (massive proliferation and activation of macrophages, neutrophils, dendritic cells, mast cells and Th17 cells) and lung damage [235]. Notably, depletion of Tregs from mice infected with murine coronavirus led to increased mortality with acute encephalitis, underlining the protective nature of Tregs during acute coronavirus infection [236]. Recently, two patients with ARDS mediated by COVID-19 were treated with ex vivo expanded allogeneic Tregs, resulting in symptom improvement, rapid decrease in inflammatory markers (IL-6, IL-12, TNF-α, IFN-γ) and no adverse effects related to the treatment [237]. Treatment of SARS-CoV-2-induced ARDS by allogeneic ex vivo expanded Tregs is evaluated in clinical trials NCT04468971 and NCT05027815.

## 7. Conclusions

In this review, we discussed the pathology of SARS-CoV-2 infection, main pitfalls and therapeutic strategies and also the numerous data highlighting the important role of CD4^+^ and CD8^+^ virus-specific T cells during COVID-19. We have also considered quantitative aspects and outlined possible treatment approaches which might still have some limitations. The cellular part of immune response against SARS-CoV-2 is characterized mainly by antiviral T cells with a broad repertoire, being detectable for more than 1 year after initial infection. In contrast to antibody generation, the protective effect of T cell response is largely also maintained toward viral variants of concern. As T cells play critical roles in the clearance of SARS-CoV-2 and protection from development of severe COVID-19, immune suppressed individuals have a higher risk for severe SARS-CoV-2 infection. This population as well as COVID-19 patients with inadequate endogenous T cell responses may benefit from adoptive transfer of allogeneic, SARS-CoV-2-specific T cells isolated from convalescent individuals. Adoptive T cell therapy is being explored as a preventative or therapeutic adjunctive therapy against SARS-CoV-2, with very promising results as enriched SARS-CoV-2-specific T cells seems to maintain proliferative capacity and cytotoxic potential toward target cells. Therefore, enriching antiviral memory T cells and depletion of potentially alloreactive naive T cells alongside precise consideration of partial HLA class matching between donor and recipient, with subsequent adoptive transfer of SARS-CoV-2-specific T cells should provide an exceptional and safe treatment option for COVID-19 patients at risk of severe disease. Infusion of SARS-CoV-2-specific T cells could be a life-saving strategy for severe SARS-CoV-2-infected patients.

## Figures and Tables

**Table 1:** SARS-CoV-2-specific T-cell clinical trials for treatment or prevention of severe COVID-19.

Clinical Trial	Therapy	Population Eligibility	Study Design	Phase of Clinical Trial	Estimated Admission	Blood Disorder Exclusion
NCT04457726	Allogeneic CSTs	1 to 90 y SARS-CoV-2 RT-PCR 1 within 72 h of enrollment	Arm A: severe COVID-19 Arm B: mild to moderate COVID-19 with high risk of progression to severe disease based on age and/ or underlying comorbidity	2/2 (recruiting)	18	Not excluded unless receiving 0.5 mg/kg/d steroids
NCT04765449	Partially HLA-matched banked allogeneic CSTs	≥18 y SARS-CoV-2 RT-PCR 1 High risk of severe disease based on age and/or underlying comorbidity No supplemental O2 requirement	Arm A: Treatment with CSTs Arm B: No available HLA-matched product, monitored at home and may receive any standard of care treatment for COVID-19	1 (recruiting)	24	Included: chemotherapy for malignancy within the prior 24 mo Excluded: prior allogeneic HSCT or solid organ transplant; current chemotherapy, radiation, and/or immunosuppressive drug regimen
NCT04742595	Partially HLA-matched banked allogeneic CSTs	≥18 y Immunocompromised with cancer SARS-CoV-2 RT-PCR 1 within 2 wk of enrollment Presence of respiratory symptoms	Patients receive CSTs on day 1 and treatment may repeat every 14 d at investigators’ discretion.	Early 1 (recruiting)	16	No (required for inclusion)
NCT04762186	Allogeneic CSTs	Maximum 14 d between symptom onset and enrollment WHO score 5 or 4 with one additional risk factor for progression	Phase I: dose finding phase Phase II: randomized pilot study comparing CST treatment at dose determined in phase I to current institutional standard of care treatment for COVID-19	1/2 (recruiting)	12	No (inclusion criteria as risk factor for severe disease)
NCT04896606	Family derived HLAmatched Allogeneic CSTs	18 to 65 y SARS-CoV-2 RT-PCR 1 Hospitalized for mild to moderate COVID-19 disease Risk of progression based on underlying comorbidity HLA-matched family related donor with recent SARS-CoV-2 infection at least 10 d from symptom onset available	Experimental arm: Patients receive family donor derived CSTs up to five times every 2 wk along with standard of care. Active Comparator: Standard of care alone.	2/2 (recruiting)	50	No (inclusion criteria as risk factor for severe disease)
NCT05141058	HSCT donor-derived allogeneic CSTs	≥12 y and <80 y ≥28 d and <4 wk after allogeneic HSCT SARS-CoV-2 RT-PCR negative	Arm A: Adults (≥18 to <80 y) will receive a single infusion of CSTs at escalating doses for prophylaxis against SARS-CoV-2 infection. Arm B: Children (≥12 and <18 y) will receive a single infusion of CSTs at escalating doses for prophylaxis against SARS-CoV-2.	2/2 (recruiting)	24	No (required for inclusion)
NCT04351659	Blood donation from convalescent donor	21 to 65 y history of COVID-19 with documented positive test for SARS-CoV-2 in the past; recovered from COVID-19 and is now suitable for blood donation, fulfilling all standard blood donor criteria; Negative test for SARS-CoV-2 currently	Patients receive blood donation from convalescent donor (1 unit)	1 (recruiting)	8	Yes (Do not meet the standard criteria for blood cell donation)
NCT05447013	CoV-2-STs generated from COVID-19recovered donors	18 to 80 yHospitalized patients, SARS-CoV-2 PCR positive, within 8 days from the onset of the symptoms who have: Pneumonia or/and SatO2 ≤ 94% on room air or/and respiratory rate ≥ 24 breaths/min AND Lymphopenia CD3+ ≤ 650/μL or/and ALC ≤ 1000/microl AND Increased values of D-dimers (≥2Χ) or/and ferritin (>1000 ng/mL) or/and CRP (≥3Χ) or/and LDH (≥2Χ)	Experimental: For Phase II: Arm A Standard of care (SOC) and Coronavirus-specific T cells (CoV-2-STs) Active Comparator: For Phase II: Arm B Standard of care (SOC)	2/2 (recruiting)	182	No

Source: https://clinicaltrials.gov/ct2/home (accessed on 15 August 2022).

## Data Availability

Not applicable.

## Abbreviations

ADAM17	A disintegrin and metalloproteinase 1
AdV	adenovirus
Ang	angiotensin
APC	antigen-presenting cell
ARDS	acute respiratory distress syndrome
ATII	alveolar epithelial type II
AT1R	angiotensin II receptor type 1
BALF	bronchoalveolar lavage fluid
BTLA	B- and T-lymphocyte attenuator
CAR	chimeric antigen receptor
CCS	cytokine capture system
CD25	cluster of differentiation 25
CD4	cluster of differentiation 4
CD8	cluster of differentiation 8
CMV	cytomegalovirus
COVID-19	coronavirus disease 2019
CST	SARS-CoV-2-specific T cell
CTLA-4	cytotoxic T-lymphocyte–associated antigen 4
dsRNA	double-stranded RNA
EBV	Epstein-Barr virus
EMA	European Medicines Agency
FDA	The United States Food and Drug Administration
FOXP3	forkhead box-p3
G-CSF	granulocyte colony-stimulating factor
GvHD	graft-versus-host disease
HLA	human leukocyte antigen
HSCT	hematopoietic stem cell transplantation
IFN	interferon
IL	interleukin
IL-2R	interleukin 2 receptor
IL-10R	interleukin 10 receptor
IRF	interferon regulatory factor
LAG-3	lymphocyte-activation gene 3
MAL	MyD88 adaptor-like protein
MasR	Mas receptor
MERS-CoV	Middle East respiratory syndrome coronavirus
MHC	major histocompatibility complex
MIS-C	multisystem inflammatory syndrome in children
MSC	mesenchymal stem cell
MyD88	myeloid differentiation primary response protein 88
N protein	nucleocapsid protein
NF-κB	nuclear factor kappa B
NK cell	natural killer cell
NKG2A	CD94/NK group 2 member A
ORF	open reading frame
PBMCs	peripheral blood mononuclear cells
PD1	programmed cell death 1 receptor
RAS	renin–angiotensin system
RNA	ribonucleic acid
S protein	spike protein
SARS-CoV	severe acute respiratory syndrome coronavirus
SARS-CoV-1	severe acute respiratory syndrome coronavirus 1
SARS-CoV-2	severe acute respiratory syndrome coronavirus 2
scFv	single chain variable fragment
ssRNA	single-stranded ribonucleic acid
TCR	T cell receptor
TGF-β	transforming growth factor beta
Tim-3	T cell immunoglobulin and mucin-domain containing-3
TIR	Toll/interleukin receptor
TLR	Toll-like receptor
TMPRSS2	transmembrane serine protease 2
TNF-α	tumor necrosis factor α
TRAM	TRIF-related adaptor molecule
Treg	regulatory T cell
TRIF	TIR domain-containing adaptor-inducing interferon-β
VST	virus-specific T cell
